# The effect of erythropoietin on healing of obstructive vs nonobstructive left colonic anastomosis: an experimental study

**DOI:** 10.1186/1749-7922-2-13

**Published:** 2007-05-15

**Authors:** Munevver Moran, M Mahir Ozmen, A Polat Duzgun, Riza Gok, Nurten Renda, Selda Seckin, Faruk Coskun

**Affiliations:** 1 Department of Surgery, Ankara Numune Teaching and Research Hospital, Ankara, Turkey; 2 Department of Pathology, Ankara Numune Teaching and Research Hospital, Ankara, Turkey; 3 Department of Biochemistry, Hacettepe University Medical School, Ankara, Turkey

## Abstract

**Background:**

Anastomotic leakage is an important problem following primary resection in the left colon and is even more prominent when obstruction is present. We aimed to evaluate the possible effects of erythropoietin on the healing of anastomosis under both obstructive and non-obstructive states.

**Methods:**

Forty male Wistar albino rats were divided into four groups. In group I, two cm left colonic resection and primary anastomosis were done. In group II, left colon were completely ligated and 24 hours later animals were re-operated for segmental resection. The same procedures were performed for rats in group III and IV in respect to group I and II and, 500 IU/kg a day erythropoietin were given in the latter two groups for seven days. For the quantative description of anastomotic healing mechanical, biochemical and histopathological parameters were employed on the seventh day and the animals were sacrificied.

**Results:**

Although erythropoietin had positive effects on bursting pressure in group IV when compared to group II, it has no effect in group III. Despite the increased tissue hydroxyproline levels in group IV, erythropoietin failed to show any effects in group III.

Erythropoietin had positive effects on neovascularization, fibroblast proliferiation and storage of collagen in group IV.

**Conclusion:**

We failed to find any direct and evident effects of erythropoietin on healing of left colonic anastomosis. On the other hand, erythropoietin might prevent negative effects of obstruction on healing.

## Background

Dehiscence of intestinal anastomosis remains a major complication after gastrointestinal tract surgery. The anastomotic dehiscence and leakage that occurs as a result of it may lead to high rates of morbidity and mortality. The risk of anastomotic leakage is high in large bowel surgery in contrast with surgery of the small bowel [1,2]. Various factors including surgical technique, the patient's nutritional state, the localization (left colon or right colon) and type of operation, the patient's age, the presence of obstruction and whether the operation was elective or emergency, which may effect the success of anastomotic healing have been studied [1-3].

Normal wound healing and tissue repair are controlled by a series of regulatory peptides which are released in response to specific stimuli and interact in a refined and coordinated manner. These peptides or growth factors have both local and systemic affect on cells [4,5]. One of the growth factors that are thought to have a positive effect on the wound healing process is erythropoietin which is a haematopoietic growth factor [6-10]. In an experimental study that examines the effect of erythropoietin (EPO) on left colonic anastomotic healing, administration of EPO appears to have beneficial positive effects on healing rate and breaking strength of large bowel anastomoses in rats [9,10]. There is no study dealing with the effects on healing after obstructive states.

Our aim was to investigate the possible effects of EPO on the healing of experimental left colonic anastomosis under both obstructive and non-obstructive states.

## Methods

### Experimental animals

The Study was approved by the animal ethics committee of Ankara University.

All the rats were obtained from the same breeding centre and were placed in a temperature-controlled environment (Ankara University Medical School Experimental Study Centre). The rats were fed with standart rat chow diet before the operation. No preoperative preparation or fasting period was tried. The rats were operated under general anaesthesia using an intramuscular injection of 35 mg/kg (*50 mg/ml flagon*) ketamin hydrocholoride (*Ketalar*^®^, *Parke Davis, Levent-İstanbul, Turkey*,) and 2 mg/kg xylasin (*Rhompun*^®^, *Bayer Türk Kimya, Şişli-İstanbul, Turkey*).

### Surgical procedure

Forty male Wistar albino rats weighing 200–250 g were divided into four groups of ten animals, three experimental and one control. Through a 3 cm midline laparotomy, the left colon was found and mobilised and a two cm segmental colonic resection and primary anastomosis was performed just two cm proximal to the peritoneal reflection both in group I and III.

The left colon was completely ligated at two cm above the peritoneal reflection using 5/0 polypropylene in group II and IV. 24 hours later animals were reoperated and a two cm segmental colonic resection and primary anastomosis was performed.

All anastomosis were performed using interrupted 6/0 polypropylene sutures, (*Prolene, Ethicon, UK*) in a single-layer, end to end and extramucosal manner. The abdomen was closed with interrupted 3/0 silk sutures (*Dogsan, Trabzon, Turkey*).

**Figure 1:**
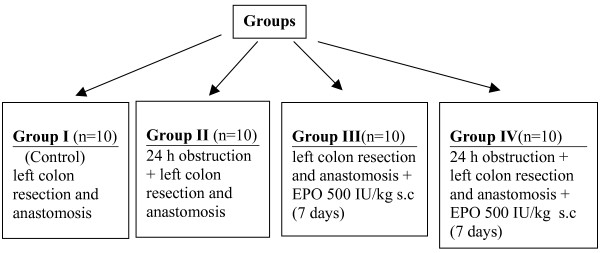
Study groups.

Animals in Group III and IV were given 500 IU/kg/day of recombinant erythropoietin (*Eprex*^®^, *Santa-Farma, Sweden*) subcutaneously for seven days following the operation. Rats in Group I and II were given isotonic sodium chloride injection subcutaneously for seven days following the operation. Study design is shown in figure 1.

Rats were allowed to have free access to water alone during the first postoperative 12 h period and fed regularly with standard chow afterwards. No antibiotics were given.

### Bursting pressure measurement

Seven days after surgery all rats underwent re-laparotomy under general anaesthesia for the determination of the *in vivo *bursting pressure(BP) prior to the death (by cardiac puncture) without interruption of the normal mesenteric blood supply or adhesions to the anastomosis using the modification of the technique described by Jiborn et al [11,12]. Anastomotic bursting pressure (ABP) was measured by passing a catheter per *anum *up to the area of anastomosis. The lumen of the colon was cleaned of feacal content by gentle wash-out with saline. Without disturbing the adhesions, the bowel (2 cm above and below the anastomosis) was tied with a 0 silk ligature. The distal catheter was connected via a pressure transducer to recorder (*Datex-Ohmeda CS/3, Helsinki, Finland*). The bowel was infused with a continuous flow of physiological saline (1.5 ml/min). The pressure in the bowel was monitored during injection and the bursting pressure (mmHg) was taken as the maximum pressure achieved during the injection phase.

After sacrification, the anastomotic site was resected and divided into two parts vertically. One used for hydroyproline measurement and the other placed in 10% formaline for histopathological examination.

### Tissue hydroxyproline measurement

Quantification of collagen in enteric anatomosis is synonymous with quantification of hydroxyproline, an amino acid unique to collagenous proteins in most tissues. Stored tissues were cleared from anastomotic materials. Approximately 50 mg of tissue was taken from each sample and colorimetric assay method was used for determination of hydroxyproline [13]. The principal of the method was the hydrolysis of the tissue specimen with 6N hydrochloric acid with the formation of free amino acids from proteins. The results were calculated as micrograms(μg) of hydroxyproline per milligram(mg) of wet tissue weight.

### Histological evaluation

After being stained with haematoxylin and eosin, colonic tissues and anastomosis were examined under light microscopy and were graded in a blind fashion, using a modified 0 to 4 numerical scale by Ehrlich and Hunt (Table 1) [14,15] The evaluated parameters were inflammatory cell infiltration, fibroblast ingrowth, neovascularization and collagen deposition. Each parameter was assessed individually using the numerical scale.

**Table 1:** Histological Grading Scale*

0 : No evidence
1+ : Occasional evidence
2+ : Light scattering
3+ : Abundant evidence
4+ : Confluent cells or fibers

The following parameters were each assessed individually: inflammatory cell infiltration, blood vessel and fibroblast in growth, and collagen deposition.

*Modified from [14]

### Statistical analysis

Data was stored and evaluated using SPSS 9.0 for Windows. All parameters were expressed as mean ± standard error of the mean (SEM). Statistical comparisons were made by using one way ANOVA and Tukey Post Hoc Tests. A p value of less than 0.05 was considered as significant.

## Results

During this experimental study, 7 rats died and new ones were included into the study. There was no spontaneous anastomotic dehiscence, intraabdominal abscesses, or other infection. All data is shown in Table 2.

**Table 2:** Mean(SEM) values of the parameters in all groups

**Groups**	**Group 1**	**Group 2**	**Group 3**	**Group 4**
**No of nonsurvivors**	2	1	1	3
**Bursting Pressure (mmHg)**	200 (5.00)	176.6 (2.30)	205 (15.23)	198.6 (2.19)
**Hydroxyproline level (μg/mg)**	1.74 (0.19)	1.63 (0.27)	1.91 (0.40)	2.52 (0.40)
**Inflammation**	2.6 (0.5)	4 (0)	3.5 (0.7)	3.8 (0.4)
**Neovascularization**	2 (0)	1.6 (0.5)	3.8 (0.4)	2.6 (0.5)
**Fibroblast ingrowth**	4 (0)	1.8 (0.8)	4.(0)	3.8 (0.4)
**Collagen Deposition**	2 (0)	1.8 (0.4)	2.6 (0.5)	2.6 80.5)

### The comparison of bursting pressures

Bursting pressures was differed significantly between the groups **(p < 0.05)**. When the bursting pressures of the control group and the others were compared, the highest value was observed in Group III (resection+anastomosis+EPO) and the lowest value was observed in Group II (obstruction+resection+anastomosis).

When we compared the results of the groups in which the same surgical intervention was performed (groups I-III, II-IV) but one in the group was given EPO and the other not, there was no statistical difference between groups I and III **(p > 0.05) **but a statistically significant difference was found between groups II and IV **(p = 0.03)**. EPO shoved positive sign of increase in the bursting pressure after obstruction. The mean bursting pressure by groups are shown in table 2.

### The comparison of tissue Hydroxyproline levels

In the groups in which EPO was given (Groups III-IV), the tissue HPO levels were higher than in the groups which were not given EPO (Groups I-II) **(p = 0.002)**. The lowest HPO levels were measured in Group II which was an obstruction-resection group. And the highest HPO levels were measured in Group IV which was given EPO after obstruction-resection. In the case where obstruction was performed, EPO increased tissue HPO levels in a more significant way. Although the HPO levels were higher in groups which EPO was given (Group III), when compared to the control group, there was no significant difference between these groups **(p > 0.05)**. The mean tissue Hydroxyproline levels of the groups are shown in table 2.

### Histological findings

In all the groups in respect to the control group, inflammatory cell infiltration was observed in a more intense way and the difference between the groups were statistically significant **(p = 0.001)**. In Groups II, III, and IV there was no significant difference in inflammatory cell infiltration **(p > 0.05)**.

There was a significant difference in the neovascularization between the groups which were given and not given EPO. In the resection+anastomosis group in which EPO was given (Group III), neovascularization was observed in an intense way in respect to all the other groups and this difference was significant **(p = 0.001)**. Futhermore, in the obstruction+ resection+anastomosis group in which EPO was given (Group IV), neovascularization was observed more in respect to Group II in which EPO was not given and this was significant **(p = 0.001)**

In Groups I, III and IV, fibroblast proliferation was observed equally and statistically insignificant **(p > 0.05)**. In Group II, in which obstruction+anastomosis was performed, fibroblast proliferation was observed lower than the other groups and the difference was statistically significant **(p = 0.003)**. It has also been observed that the obstruction had a negative effect on tissue fibroblast proliferation and this effect disappeared after EPO injections. In spite of this, EPO did not have a significant effect on anastomosis which was performed without obstruction in respect to fibroblast proliferation.

Although the storage of collagen was equal and higher in groups in which EPO was given (Group III and Group IV), when compared to the control group, there was no significant difference **(p > 0.05)**. In contrast, when Group III and Group IV were compared with Group II which had the lowest storage of collagen, the difference was significant **(p = 0.02)**. In this case, it was observed that the decreased storage of collagen after resection increased positively with the administration of EPO and it was significant in the obstruction group.

## Discussion and conclusions

Insufficiency of intestinal anastomosis remains the most important cause of morbidity and mortality after gastrointestinal tract surgery. Despite the increased risk of leakage after emergency procedure at all sides of the intestine, the occurrence of anastomotic leakage appears more frequently during colonic operations with high morbidity and mortality.

Various factors have been shown to effect healing of anastomosis [1-3].

One of the growth factors thought to have a positive effect on the wound healing process is erythropoietin which is a haematopoietic growth factor. Erythropoietin(EPO) is a glycoprotein with a true hormonal structure which is located in the alpha-globulin fraction of the plasma and has a molecular weight of 46.000 kilodaltons. Like growth hormone(GH), it is also a member of the hematopoietic super family which consists of GH, EPO, granulocyte and macrophage colony-stimulating factor, interleukin 3 [IL-3], IL-4, IL-6, IL-7. All of them have similarities in their receptor structure [16,17]. It is likely that cross-reactivity between certain growth factors may exist. EPO is produced mainly in the kidneys and to a lesser extent in the liver [18]. Circulating EPO binds to EPO receptors on the surface of erythroid progenitors resulting in the replication and maturation to functional erythrocytes [19]. In studies, its receptors have also been found in the kidneys, liver, brain, the intestines, bone marrow and cardiomyosites [5-10,20,21]. It also shows an anabolic effect on wound healing by affecting other growth factor receptors thus increasing fibroblast proliferation, collagen deposition, endothelial cell proliferation (angiogenesis) and the manufacture of extracellular matrixes. It also has a trophic affect on gastrointestinal growth and development and increases cell turnover and cell migration [8]. Recombinant EPO has also been used in patients awaiting elective surgery to increase their packed red blood cell volume [22].

In an experimental study which was done by Fatouros that examines the effect of EPO on colonic anastomose healing, it has been established that, among the rats which were given EPO for 15 days preoperatively and 7 days postoperatively, the breaking strength of primary left colonic anastomosis increased by 37% [9,10]. In histological examination on the anostomotic area, a smaller number of inflammatory cells have been observed in the EPO group, when compared to the control group. Despite this, more fibroblast reaction and angiogenesis was observed in the EPO group when compared to the control group.

In order to investigate the effect of EPO on wound healing in resection and anastomosis after colonic obstruction, a new group was added and EPO administration was applied for 7 days postoperatively. The healing of anastomosis was evaluated by using mechanical bursting pressure measurement, the tissue hydroxyproline level, and the collagen storage score during histological examination.

Although tissue hydroxyproline and bursting pressure levels in Group III were both higher than the control group no significant difference was found in groups without obstruction. Using these results we might say that EPO could have positive beneficial effects after primary left colonic anastomosis however, in comparison to Fatouros' study this improvement was not significant.

The results of the histological evaluation show that the histological inflammation score of the groups in which EPO was given was significantly higher in respect to the control group and there was no difference between Group I and III (EPO was administered in Group III but not in Group I) in terms of fibroblast proliferation scores. In addition to this, the high level of the neovascularization score in the groups which EPO was given supported the previous findings that EPO increased neovascularization. The speed of neoangiogenesis in the EPO group could suggest the presence of EPO receptors in the endothelial cells. These receptors have previously been found in humans like in rats [21].

The collagen score of Group III (in which erythropoietin was given) was higher than Group I with no statistical significance. Similar magnitude of increase also seen in the hydroxyproline level and the breaking strength of the anastomosis which led us to conclude that although erythropoietin definitely has positive effects on the anastomosis of the left colon, but these effects are not significant. The difference between our study and the previous study might partly be attributed to the timing and the dosage of EPO as they used it for a longer period[9,10].

It has been stated in most of the published studies so far that in the healing of the primary anastomosis performed due to left colonic obstruction, more problems appeared and morbidity and mortality increased clinically when compared to right colon. It has also been stated that the reasons for this problem, besides the operations which have been performed in emergency and without preparing the patient adequately after obstruction, include various causes like the faecal load of the unprepared colon, which is the most important one [23,24]. It has been shown that intraoperative colonic lavage and decompression have a positive effect on anastomotic healing via decreasing faecal load in the surgical treatment of left colonic lesion with obstruction [25]. Therefore, in the groups with left colonic obstruction, the faecal particles at the anastomosis area were cleaned by rubbing in the present study. Preoperative bowel preparation and another decompression was not performed and the rats were not left hungry for the reoperation next day. In that respect, one might say that the increased intraluminal faecal load due to obstruction would have a negative effect on anastomotic healing. However, in the group EPO was not given (GroupII) anastomotic bursting pressure and tissue hdroxyproline levels were found to be significantly lower as compared to the control group. Also, neovascularisation, fibroblast proliferation and collagen storage scores were also found lowest in Group II during histology. Whereas in Group IV (obstruction+ resection+anastomosis+EPO) the results were completely different: the average bursting pressure and tissue hydroxyproline levels were significantly higher than group II and these results were similar in the nonobstructed groups I and III. The beneficial effects of EPO administration in anastomotic healing of the obstruction group was also seen in the histological findings. In Group IV, the average neovascularization score was higher than in Group II. However, the fibroblast proliferation parameter in group IV, which was similar to the control group (p > 0.05), was significantly better than Group II (p < 0.05). The mean collagen storage score in Group IV was higher than in Group II and the control group. In the obstruction group EPO administration was found to be beneficial.

The question that should be asked is why and how erythropoietin has a positive effect on colonic anastomosis with obstruction but not on colonic anastomosis without obstruction.

In this study; the Group II had the worst anastomotic healing. In this group neovascularization, fibroblast proliferation and collagen synthesis were all found to be significantly lower but inflammation was significantly higher than the others. In both the obstruction + anastomosis groups the inflammation rate was similar, but neovascularization, fibroblast proliferation and collagen storage level were significantly better.

The inflammation period is the initial phase of the healing process. The most important factors which prolong the inflammation period are the presence of foreign body and bacterial inoculation in the wound. When there is an infection in the wound, completion of the healing process is impossible.

Bacterial overload and increased faecal content in the proximal part of the obstructed colon, local contamination during resection and anastomosis might all be responsible for these higher rates of inflammation and complications [23-29].

These observations indicate that the positive effects of EPO on growth factor in wound healing must be seen in higher inflammation rates. Thrombocytes, lymphocytes and macrophages are the cells that take place in the inflammatory cascade. As we know, these cells release lots of growth factors that are polipeptides and have mitogenic, chemotactic and cell movement stimulant functions. They attack target cell receptors. It is also known that they activate their functions with cross reactions at receptor level [30-32]. EPO receptors have been found in rats and human endothelial cells [22]. It is believed that EPO interacts with endothelial cell receptors and stimulate healing process by increasing neovascularization. The significant observation of the increased neovascularization score in the groups which have been given EPO supports the previous findings [9,10]. Neovascularisation alone does not explain the positive effects of EPO on anastomotic healing in the resection+anastomosis group after colonic obstruction.

In conclusion, we failed to find any direct and evident effect of erythropoietin on healing of left colonic anastomosis in the present study. On the other hand, it was found that erythropoietin might prevent negative effects of obstruction on healing by increasing bursting pressure and tissue hydroxyproline levels.
